# Complexity of the MSG gene family of Pneumocystis carinii

**DOI:** 10.1186/1471-2164-10-367

**Published:** 2009-08-07

**Authors:** Scott P Keely, James R Stringer

**Affiliations:** 1 Department of Molecular Genetics, Biochemistry and Microbiology, University of Cincinnati College of Medicine, Cincinnati, Ohio, 45220, USA

## Abstract

**Background:**

The relationship between the parasitic fungus *Pneumocystis carinii *and its host, the laboratory rat, presumably involves features that allow the fungus to circumvent attacks by the immune system. It is hypothesized that the major surface glycoprotein (MSG) gene family endows *Pneumocystis *with the capacity to vary its surface. This gene family is comprised of approximately 80 genes, which each are approximately 3 kb long. Expression of the MSG gene family is regulated by a cis-dependent mechanism that involves a unique telomeric site in the genome called the expression site. Only the MSG gene adjacent to the expression site is represented by messenger RNA. Several *P. carinii *MSG genes have been sequenced, which showed that genes in the family can encode distinct isoforms of MSG. The vast majority of family members have not been characterized at the sequence level.

**Results:**

The first 300 basepairs of MSG genes were subjected to analysis herein. Analysis of 581 MSG sequence reads from *P. carinii *genomic DNA yielded 281 different sequences. However, many of the sequence reads differed from others at only one site, a degree of variation consistent with that expected to be caused by error. Accounting for error reduced the number of truly distinct sequences observed to 158, roughly twice the number expected if the gene family contains 80 members. The size of the gene family was verified by PCR. The excess of distinct sequences appeared to be due to allelic variation. Discounting alleles, there were 73 different MSG genes observed. The 73 genes differed by 19% on average. Variable regions were rich in nucleotide differences that changed the encoded protein. The genes shared three regions in which at least 16 consecutive basepairs were invariant. There were numerous cases where two different genes were identical within a region that was variable among family members as a whole, suggesting recombination among family members.

**Conclusion:**

A set of sequences that represents most if not all of the members of the *P. carinii *MSG gene family was obtained. The protein-changing nature of the variation among these sequences suggests that the family has been shaped by selection for protein variation, which is consistent with the hypothesis that the MSG gene family functions to enhance phenotypic variation among the members of a population of *P. carinii*.

## Background

*Pneumocystis carinii *is a fungal microbe that is found in the lungs of laboratory rats [1-6]. *P. carinii *appears to be specific to rats because it is not found in other species of mammals and fails to establish itself when introduced into immunodeficient mice [7], which have their own species of Pneumocystis, called *P. murina *[8]. *P. carinii *is morphologically and phylogenetically closely related to *P. murina*, both of which are somewhat less closely related to the human pathogen, *Pneumocystis jirovecii*, which causes Pneumocystis pneumonia in individuals with impaired immune function, such as patients suffering from Acquired Immunodeficiency Syndrome (AIDS) [3,9-15]. *P. carinii *and *P. murina *can cause pneumonia in their hosts, rats and mice, respectively, if these host animals lack a robust immune system [16-19].

While *P. carinii *can cause disease in the absence of a normal immune system, rats that lack such a system are probably not its normal ecological niche. It has been established that *P. carinii *organisms can persist for months in rats that are immunologically normal [20]. Normal laboratory rats are often colonized by *P. carinii *and show no obvious ill effects [5,6]. Likewise, *P. murina *appears to be able to inhabit normal mice [16,17,21-23]. By analogy, *P. jirovecii *would be expected to make its home in normal humans, and data showing colonization of healthy people by *P. jirovecii *are accumulating [24-33].

None of the species of *Pneumocystis *that have been studied have been observed to proliferate much outside of the airway of the mammalian host in which they are found, and Pneumocystis DNA is very scarce in environments apart from mammals [34-38]. Thus, *Pneumocystis *species exhibit three features suggesting that they are obligate parasites of mammals: 1) They are extremely scarce outside of the mammalian lung. 2) They have fastidious growth requirements. 3) They can colonize immunocompetent hosts.

Parasites employ various methods to survive in the face of host defenses. One such method is programmed antigenic variation, which allows a population of parasites to quickly produce an organism whose surface differs from that of the others in the population. The VSG antigenic variation system in the protozoan parasite *Trypanosoma brucei *illustrates how gene families can be used to create phenotypic diversity within a population of eukaryotic parasites [39-46]. There are thousands of different VSG genes in the *T. brucei *genome [47]. These genes tend to be clustered together near telomeres. Only one VSG gene is transcribed in a given cell. The gene that is expressed changes frequently enough to make it probable that the host immune response, which is directed against the version of VSG present on the majority of parasites, does not destroy all of the parasites in the host. Changing which gene is expressed in *T. brucei *is often accomplished via DNA recombination, which alters an expressed VSG gene by replacing some or all of its DNA with DNA from a silent VSG gene [46,48,49].

The *P. carinii *MSG (Major Surface Glycoprotein) gene family is much smaller than the *T. brucei *VSG gene family, but exhibits structural and functional features similar to it. The *P. carinii *genome contains approximately 80 MSG genes, which are located at the ends of each of 17 chromosomes [50-55]. Pairwise comparisons of eleven complete *P. carinii *MSG showed that they are between 5 and 19% divergent, but share a number of features including a length of approximately 3 kb, a lack of introns and the presence of an invariant 5' sequence element called the CRJE, which is discussed further below [55]. Other short invariant sequence elements reside at multiple locations within the bodies of the 11 fully sequenced MSG genes, which tended to be least variable at their 3' ends. Most of the 11 genes have been shown to be members of gene clusters containing up to 3 MSG genes. The genes within a cluster were as different from one another as they were from genes in different clusters, suggesting that selection and or recombination has driven rapid diversification of *P. carinii *MSG genes [55].

MSG genes have been described in five other species of Pneumocystis, including the three that have received a species name other than " carinii", *P. murina *(found in the laboratory mouse) [56], *P. wakefieldiae *(found in the laboratory rat) [57] and *P. jirovecii *(found in human beings) [58,59]. MSG sequences have also been reported from two additional presumptive Pneumocystis species (one from ferrets and one from a macaque) that do not yet have their own species name [60,61].

Studies on restriction enzyme fragment length polymorphism have shown that there is considerable variation in the MSG gene families present in *P. jirovecii *organisms found in different human beings [59]. These finding are consistent with the idea that MSG genes evolve rapidly. Compared to *P. jirovecii *MSG genes, neither *P. carinii *nor *P. murina *MSG genes families exhibited much variation when studied by restriction enzyme analysis [59]. Nevertheless, it is possible that MSG gene families are evolving relatively quickly in each species of *Pneumocystis*, and that the more limited MSG diversity seen in *P. carinii *and *P. murina *reflects the isolation of laboratory rodents, a practice that would be expected to limit exposure to the populations of *Pneumocystis *that live in wild rodents. While keeping rodents in vivaria keeps exogenous microbes out, it would also tend to trap any endogenous parasites. It is common to find *P. carinii *at low levels in laboratory rats that have not been deliberately exposed to the fungus, indicating that a particular population of *P. carinii *can propagate within colonies of laboratory rats [5]. Therefore, the reason that *P. carinii *found in laboratory rats tend to be relatively genetically uniform may be that these microbes descended from those that were captured along with the rats that were used to establish laboratory colonies. By contrast, human beings would be expected to encounter multiple wild strains of *P. jirovecii*.

**Figure 1:**
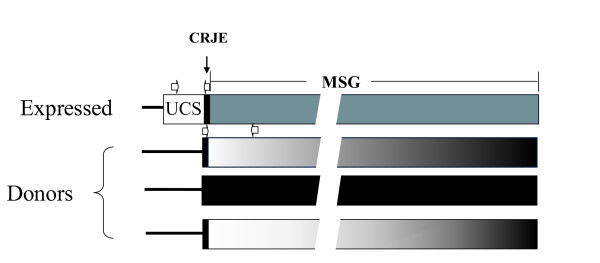
**Maps of expressed and donor MSG genes**. The expressed MSG gene is adjacent to the UCS sequence. Donor MSG genes are not adjacent to the UCS sequence, which is unique in the genome. For illustrative purposes, just three of approximately 80 donor genes are depicted. There is a copy of the CRJE, which is a 24 basepair conserved sequence, at the beginning of MSG genes, including the one attached to the UCS. The discontinuities in the MSG genes are there to indicate that the genes are not drawn to size relative to the UCS and CRJE. The open horizontal arrows show the locations and orientations of PCR primers. The two arrows above the expressed gene represent the -145 and α-CRJE primers (Additional file 3, **Table S2**). The two arrows above the first donor gene represent the CRJE and C2 primers (Additional file 3, **Table S2**).

Expression of MSG gene families has been studied primarily in *P. carinii*, where several lines of evidence indicate that a single MSG gene is transcribed in a given *P. carinii *genome at a given time. Restricted transcription is accomplished via a cis-dependent mechanism that involves a unique telomeric site in the genome called the expression site. Only the MSG gene adjacent to the expression site is represented by messenger RNA [52,53,62,63]. The MSG protein on the surface of *P. carinii *organisms has been shown to vary and to be encoded by the MSG gene that is at the expression site [64,65]. The expression site contains the UCS (Upstream Conserved Sequence), a sequence found at the beginning of messenger RNAs encoding diverse MSG proteins [62,63] (Figure 1). Immediately adjacent to and downstream of the UCS, there is short sequence, called the CRJE, which is conserved among all MSG genes, by definition [52,62,66].

CRJE stands for "Conserved Recombination Junction Element" because it could be involved in recombination events that cause the MSG gene at the expression site to change [62]. The CRJE is present both at the expression site at the junction between the UCS and the expressed MSG gene, and in MSG genes that were not attached to the expression site (donor MSG genes) (Figure 1). The location and conservation of the CRJE suggests that it could function as a target of a site-specific event, such as a double-stranded break, which would be expected to increase recombination between the expression site and a donor MSG gene. However, there is no direct evidence for such events and the role of the CRJE in recombination is still a matter of speculation. Nevertheless, the CRJE serves to identify MSG genes, which otherwise resemble MSR genes, a *P. carinii *gene family that is not regulated by the MSG expression site [66-68].

A large variety of different MSG sequences have been observed at the expression site, indicating that recombination can install DNA from various silent donor MSG genes at this locus [52,53,69]. The types and frequencies of the inferred recombination events are not clear because the fastidiousness of *P. carinii *has prevented experiments *in vitro*, and experiments in rats are complicated by their tendency to be colonized by *P. carinii*, which has limited the utility of experiments that seek to observe phenotypic or genotypic switching by introducing into a rat a small population of *P. carinii *expressing a known MSG gene [69]. An alternative approach to understanding the MSG system would be to acquire a better understanding of the gene family. If all of the genes in the family are identified, it may be possible to infer how changes are produced at the expression site. For example, if recombination completely replaces the MSG gene at the expression site with an MSG gene from a donor site, then the sequences found at the expression site will match those in the donor gene database. However, if recombination were to alter a segment of the MSG gene that is at the expression site, then there will be sequences linked to the UCS that do not exactly match any of the donor genes.

Understanding the MSG gene family at the sequence level will also aid in assessing the function of this family. If its function is to confer variability, then MSG genes will have evolved under the influence of selection for variation in the proteins they encode (positive selection), a prediction that can be tested by sequence analysis.

Sequence data pertaining to the *P. carinii *MSG gene family are available, but the vast majority of the available data has not been analyzed [70]. Analysis of these data is challenging for several reasons. First, most of the data are in the form of shotgun reads which require assembly. However, standard assembly procedures are not designed to assemble genes from gene families, and might join reads that came from different genes. A second possible complication stems from the doubtful clonality of *P. carinii *populations. The organisms used to obtain genome sequence data came from the lungs of immunosuppressed rats that had been infected by constant exposure to other infected rats [63]. This system of obtaining infected rats has been in operation for decades. Hence, more than one genetic strain of *P. carinii *could have contributed to the DNA used to obtain genomic sequences, and a given MSG gene could be represented by more than one allele. Alleles are defined as different versions of the sequence located at a particular location on a chromosome (i.e., at a genomic locus). In absence of gene flow between two populations that were genetically identical at separation, mutation will cause allelic polymorphism to arise over time. The formation of allelic polymorphism would be accelerated if selection were to favor cells that sustained mutation, as could be the case for MSG genes given their probable role in generating phenotypic variation. *P. carinii *cells are thought to be haploid, and a given haploid cell can contain only one allele at each locus. Nevertheless, two strains might contain two different alleles of a particular gene at a particular locus. Therefore, if more than one genetic strain of *P. carinii *contributed to the sequence data, then a given MSG gene could be represented by more than one allele. Assembly programs would tend to amalgamate alleles into a single consensus sequence, thereby obscuring an important aspect of the sequence data.

A third problem is posed by the presence of MSR genes in the *P. carinii *genome. MSR and MSG genes are distinct, but highly related, and analysis of sequence data must be performed in a way that avoids sequence reads from MSR genes.

In the studies described herein, the first 300 basepairs of MSG genes were selected for analysis. Although MSG genes are more than 3000 basepairs long, analyzing the first 300 bps of MSG genes offered two important practical advantages. First, this segment of an MSG gene is specifically amplifiable using the CRJE as a primer-binding site (Figure 1). Hence, this approach avoids interference from MSR genes, which lack the CRJE. Second, the 300 bp amplicons are smaller than the average sequence read available in the largest database, that of the Pneumocystis genome project. Therefore, it seemed probable that sequence reads spanning the entire 300 bps would be numerous in the database, in which case it would be possible to cover the whole family without having to rely on assembly of contigs, which is problematic when dealing with gene families. Practical advantages aside, the 5-prime ends of MSG genes are of interest because recombination events that move DNA from donor genes to the gene at the expression site may be frequent in this region. Defining the full repertoire of donor MSG genes should allow this hypothesis to be tested in the future.

## Results

### Strategy and approach for sequence analysis of the MSG gene family

Assembly of short sequence reads from a gene family can be problematic because assembly relies on sequence identity, and members of gene families tend to share bits of sequence identity. Therefore, the assembler might join sequences that represent different members of the family. While mis-joining can be minimized by increasing the number of consecutive bases that must match, doing so reduces the number of useful sequences and raises the risk of failure to join sequences that represent the same gene, but are slightly different due to errors or allelic variation. Although the *P. carinii *genome appears to be haploid, a given gene could be represented by more than one allele because stocks of *P. carinii *available for sequence analysis may have included a variety of genetic strains. In the case of *P. carinii *MSG genes, analysis is further complicated by two other factors. First, the *P. carinii *genome contains a second gene family, MSR, members of which are very similar to members of the MSG gene family [66,71,72]. Second, rats can be co-infected by a second species of Pneumocystis, *P. wakefieldiae *[73-76]. In light of these considerations, the first 300 nucleotides from MSG genes were chosen for analysis. This segment can be amplified and sequenced as a single unit that contains the CRJE, a sequence element that distinguishes MSG genes from MSR genes, and distinguishes *P. carinii *MSG genes from *P. wakefieldiae *MSG genes [75].

**Figure 2:**
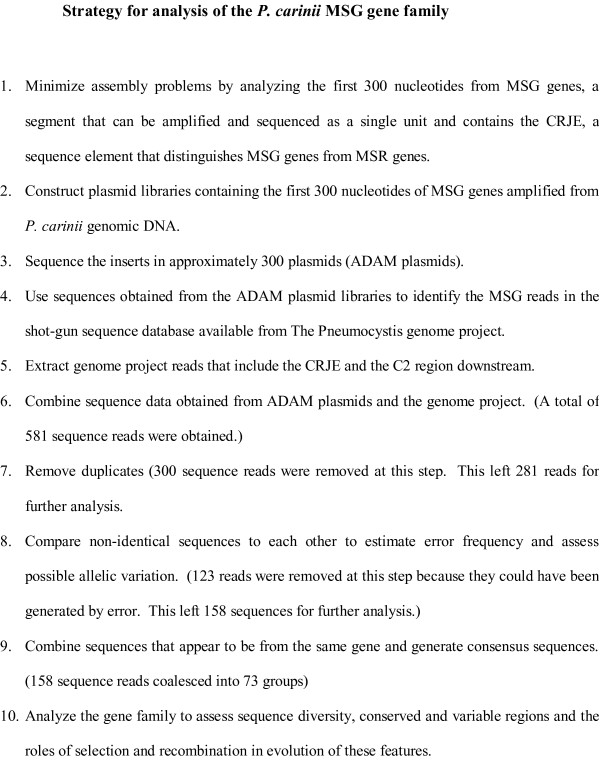
**Strategy for analysis of the *P. carinii *MSG gene family**.

The strategy for acquisition and analysis of sequence data is summarized in Figure 2. All of the sequences acquired via this strategy began with a copy of the CRJE and ended with a conserved sequence called C2 [55]. Therefore, all of the sequences overlapped by at least 300 bases.

### Comparison of all sequence reads

Combining the sequence reads found in the genome project database with the sequences obtained from ADAM plasmid libraries provided 581 sequences, which was more than 7 times as many as the 80 genes thought to be in the *P. carinii *MSG gene family [50-55]. Some sequences were present dozens of times among the 581, while other sequences were present only once. Variation in the number of times a sequence was present occurred both in data from the ADAM plasmids and in reads from the genome project.

There were 281 different sequences among the set of 581. Pairwise comparisons of the 281 non-identical sequences showed that the average number of non-identical sites between any two sequences was 56 ± 4 (approximately 19%), a value similar to that observed when 11 full-length MSG genes were compared [55] (Additional file 1). The difference-values were distributed around the mean in a manner that resembled a normal distribution, with approximately 95% of the data falling between 10% and 30%, which was within 2 standard deviations of the mean. However, pairs of sequences that differed by less than 10% were apparent in the distribution, indicating that some sequence reads were very similar to others.

**Figure 3:**
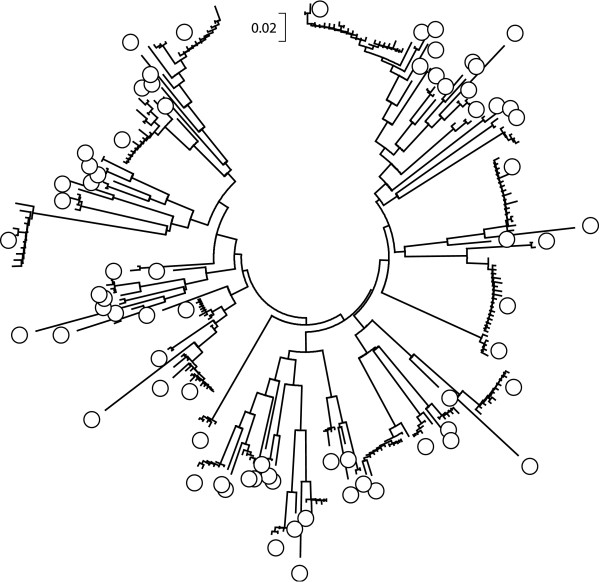
**Neighbor-Joining Tree of 281 MSG sequences**. The relationships among the 281 non-identical MSG sequences were inferred using the Neighbor-Joining method [121] in MEGA4 software [117]. Gaps were ignored. Circle symbols mark the branches containing members of the 73 core MSG sequences (see text). The tree is drawn to scale in p-distance units. The bar at the top of the tree represents a p-distance of 0.02.

Figure 3 shows a tree depicting the relationships among the 281 non-identical sequences. Again, it was apparent that some sequences were very similar to one another. Closely related sequences are expected to arise due to PCR and other errors. Therefore, the next step was to determine if error contributed to the diversity observed.

### Estimation of error in sequence reads

To assess error in the sequence data, sequence reads that were at least 99% identical were aligned. The reads in groups containing 10 or more reads were visually inspected to detect cases where a single sequence read differed from all others in the group. Such differences were scored as errors. There was one such presumptive error for every 769 bp, indicating that the error frequency per bp was 0.0013. Transitions outnumbered transversions by about 3 to 1, as expected [77]. The observed error frequency agreed with that expected from an independent estimate of PCR error, which was obtained by sequencing cloned PCR products produced by amplifying a cloned DNA fragment (data not shown). In addition, the error frequency was the same as that reported in a study on errors in sequences from Drosophila species [78].

Single errors were most common, occurring in 22.4% of the reads, while 5.8% contained two errors, and 1.7% contained three errors. These data were similar to those expected based on Poisson probability calculations, which predicted that 26.4% of the reads would contain 1 error, and 5.1% would contain two errors. However, Poisson probability predicted that only 0.7% would contain three errors. Therefore, sequences with three putative errors seemed to be more common than would be expected by chance. Nevertheless, statistical analysis of the observed and expected error data (by Chi square goodness of fit test) showed the two data sets to be statistically indistinguishable (p = 0.5).

Analysis of closely related sequence reads identified 123 reads that differed from other reads at only one position. Given that such minimal divergence could have been due to error, it seemed justifiable to restrict further analysis to the 158 reads that differed at more than one site.

### Reassessment of the number of MSG genes in the gene family

Previous data had suggested that there are only about 80 genes in the family [50,69,79-81]. Yet, the sequence data contained at least 158 genuinely different sequence reads (i.e., those that exhibited more divergence than would be expected to be produced by error). Therefore, the number of genuinely different sequences was at least twice as high as the putative number of genes in the family. This observation prompted a reexamination of the size of the MSG gene family.

**Figure 4:**
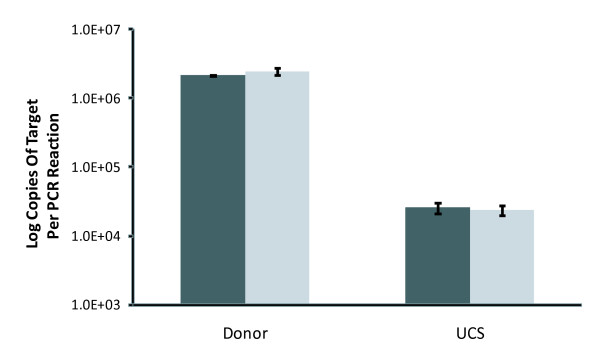
**Quantification of the number of MSG genes by real-time PCR experiments**. Donor genes were amplified using primers CRJE-RT and C1 (Additional file 3, **Table S2**). The UCS was amplified with UCS primer -145 and primer α-CRJE (Additional file 3, **Table S2**). At least three independent PCR reactions were performed with a given primer pair. The two bars of different shades show data obtained from *P. carinii *populations isolated from two different rats.

A quantitative PCR assay was developed based on the fact that all MSG genes contain a copy of the CRJE, by definition, while MSR genes do not contain the CRJE [66]. A second primer site was identified approximately 80 bp downstream of the CRJE. At this location, all of the 581 sequence reads contained a completely conserved sequence, called C1 (see section titled "Conserved and variable regions in members of the MSG gene family"). Therefore, PCR using a CRJE primer paired with a C1 primer should amplify the MSG gene family exclusively and exhaustively. The abundance of MSG targets was compared to the abundance of UCS targets. The UCS is present in one copy per haploid genome [52,53,62,63,80,82-84]. The real-time PCR results indicated that the *P. carinii *genome contains approximately 90 genes encoding MSG (Figure 4). These results were consistent with previous estimates [50,69,79,80].

### Evidence of allelic variation

Allelic variation would explain both the presence of very closely related sequences and the apparent excess of different sequences relative to the number of MSG genes in the genome. Although the *P. carinii *genome is probably haploid [85], multiple alleles could have been present among the organisms used to obtain sequence reads because the populations of organisms were not necessarily genetically homogeneous. The source of the *P. carinii *present in the rats used to isolate *P. carinii *was not well-defined because laboratory rats tend to carry *P. carinii*, which they can acquire soon after birth [6,86]. In addition, the *P. carinii *used in the preparation of the various libraries had been obtained from different rats at different points in time. Therefore, if different genetic strains of *P. carinii *exist, more than one could have been represented in the DNA used to obtain sequence data.

To examine the possibility that allelic polymorphism contributed to the diversity of MSG sequences observed, sequence reads that differed by no more than 5% were grouped. This procedure incorporated all but 31 of the original 581 sequences into 42 groups, and left 31 sequences as singletons. The groups varied in size from 49 to 2 members.

To determine if polymorphism occurred within a group, the reads were aligned and the alignment scanned visually to identify sites that varied in a way that was inconsistent with error. Given that the probability of error was 0.0013 per site, the probability that an error would occur twice at a given site was less than 2 × 10^-6^. Therefore, observation of a variant nucleotide at the same site in at least two reads in a group would seem to be due to polymorphism, rather than error. Based on this criterion, polymorphism was present among the sequence reads in 21 of the 42 groups (Table 1 and Additional file 2). Statistical analysis supported the designation of recurrent substitutions as polymorphism, rather than error. For example, reads with two or more substitutions were 5 to 28-fold more numerous than would be expected based on the error rate (p < 0.001, Chi square goodness of fit).

**Table 1:** Frequencies of putative MSG alleles in five populations of *P. carinii*

Group^a ^(no. reads)	Nucleotide position of polymorphism^b^	Haplotypes^c^	Frequency of haplotypes in 5 *P. carinii *populations^d^				
			A	B	C	D	E

1(49)	HV1, 81	1-C	15	3	1	1	5

		1-T	0	2	0	0	0

		2-C	4	15	0	0	1

		2-T	0	1	0	0	0

		3-C	1	0	0	0	0

		4-C	1	0	0	0	0

							

2 (40)	57,83,173,204	5-GTAA	6	24	0	0	0

		5-TTAA	0	2	0	0	0

		5-GCAA	0	2	0	0	0
		5-GTGA	4	0	0	0	0

		5-GTAT	2	0	0	0	0

							

3 (34)	HV1, 85, 203	4-CT	9	16	1	0	1

		4-CG	1	0	1	0	0

		6-CT	0	0	0	0	1

		6-CG	2	0	0	0	0

		5-GG	1	0	1	0	0

							

4 (32)	HV1	7	10	16	1	0	0

		8	2	0	0	0	0

		9	0	0	1	1	0

		10	1	0	0	0	0

							

5 (29)	153	11-T	5	22	0	0	0

		11-C	0	2	0	0	0

							

6 (28)	HV1, 300	12-T	4	20	1	0	0

		12-C	0	2	0	0	0

		13-T	1	0	0	0	0

							

7 (26)	193	14-A	2	22	0	0	0

		14-G	0	2	0	0	0

							

8 (24)	HV1	9	13	7	0	0	1

		7	2	0	0	1	1

9 (25)	HV1,302,313	4-AA	3	14	0	0	0

		4-GA	0	4	0	0	0

		4-AG	0	2	0	0	0

		1-AA	1	0	0	0	0

		15-AA	1	0	0	0	0

							

10 (19)	97 to 107	16	8	10	0	0	0

		16-indel	1	0	0	0	0

							

12 (18)	65,216,228,282	18-T-TA	2	11	0	0	0

		18-A-TA	0	1	0	0	0

		18-AATG	0	1	0	0	0

		18-TATA	0	1	0	0	0

		18-T-AA	0	2	0	0	0

							

13 (16)	HV1	9	1	0	0	0	0

		16	15	0	0	0	0

^a ^581 reads were assembled (maximum mismatch of 5%) into groups. Groups containing less than 16 reads are not listed.

^b ^"HV1" means that a polymorphism was seen in hypervariable region 1 (see Table 2 for sequences). Numbers in this column refer to the location of the polymorphisms that were not in HV1. Each number refers to a nucleotide site where position 1 is the A in the ATG at the beginning of the CRJE. Because the HV1 sequences varied in length, the position-numbers of polymorphisms downstream of HV1 cannot be compared between groups.

^c ^An haplotype designated as 1-C had a type 1 hypervariable region and a C at a polymorphic site located outside of HV1.

^d ^Populations A and B were the source of ADAM plasmids and Lucigen genome project reads, respectively. Populations C, D and E were smaller populations that had been analyzed in the past using the same methods as those used to produce the ADAM plasmid library.

Different versions of a group sequence were rarely seen in equivalent numbers. In 5 of the 7 groups that contained 25 or more reads (see groups 1–7 in Table 1), the majority sequence was at least 10 times more frequent than the minority sequence. The predominance of one version of a sequence within a group is inconsistent with the idea that minor sequence variation is due to the presence of very similar genes that reside at different loci, because such a situation would cause the different versions of a sequence to be seen in equal numbers, which was seldom the case.

If a polymorphism reflects allelic variation, then one population might be rich in one allele, while another allele predominates in a second population. By contrast, if two genes gave rise to an observed sequence polymorphism, then both genes will be present in all populations. In group 1, haplotype 1-C was present in 15 of the 21 reads obtained from ADAM plasmids (population A in Table 1), and in 5 of 6 plasmids from population E, but in only 3 of the 21 reads from the reads obtained from Lucigen plasmids (population B in Table 1), where haplotype 2-C predominated. The probability of observing these haplotype frequencies under the two-gene model is very low (p = 0.0004, Fisher Exact Probability Test). In group 2, nearly all of the 28 sequence reads obtained from Lucigen plasmids were haplotype 5-GTAA. However, four other haplotypes were also observed in group 2. If any of these other haplotypes came from a second gene, then the probability of observing 24 sequences with the 5-GTAA haplotype among the 28 sequences from the Lucigen plasmids is very low (p = 0.009, Fisher Exact Probability test).

### Conserved and variable regions in members of the MSG gene family

To simplify analysis of variation among the different groups of sequences observed, a consensus sequence for each of the 42 sequence groups was produced. Combining these 42 consensus sequences with the 31 singleton sequences produced a set of 73 sequences that can be considered to represent the core of the MSG gene family.

**Figure 5:**
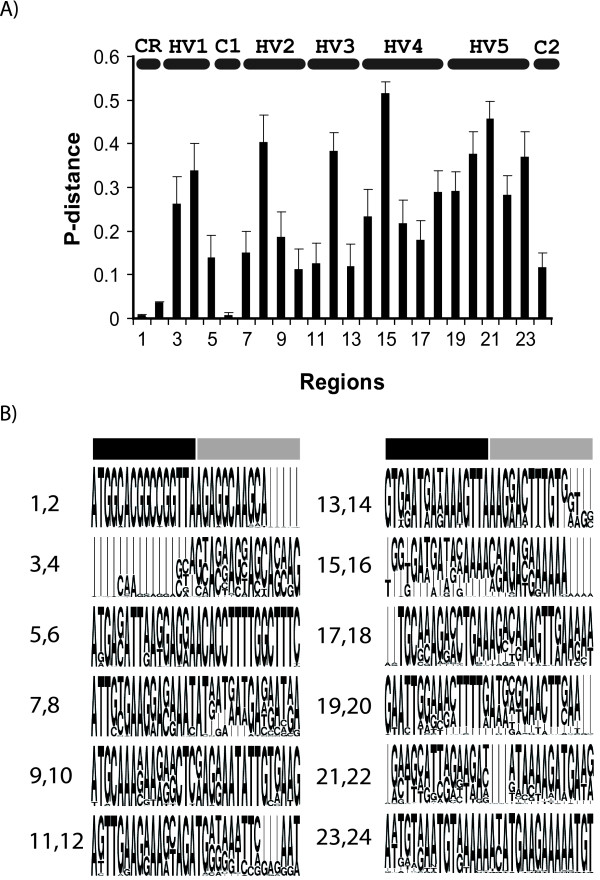
**Conserved and variable regions in MSG genes**. A) The 73 core MSG sequences were aligned. The DNA alignment was partitioned into regions containing 16 bp and average p-distances were calculated for each region using MEGA4 software [117]. The horizontal lines labeled HV1 etc, demarcate five hypervariable regions. The horizontal lines labeled CR, C1 and C2, demarcate constant regions CRJE, C1 and C2. B) A depiction of the majority and minority sequences observed. The height of a letter is proportionate to the frequency at which the base it represents was observed in the 73 aligned sequences. Thin vertical lines represent positions where INDELS occurred in the alignment. Each of the 12 blocks of sequence contains two of the twenty-four 16-base segments analyzed in panel A. The limits of each 16-base segment are indicated by the black and gray horizontal bars. The leftmost pairs of numbers correspond to the region-numbers in panel A. For example, the first block of 32 bases contains regions 1 and 2. Region 1 is covered by the black bar. Region 2 is covered by the gray bar.

Figure 5 shows where sequence variation occurred among the MSG genes represented by the 73 core sequences. Nucleotide conservation was strongest in the following three regions: (i) the first 24 basepairs, which comprise the CRJE, (ii) the region including nucleotides 80–99, which was designated conserved region 1 (C1), and (iii) the last 22 nucleotides, which corresponds to conserved region 2 (C2) [55,69]. (Because the data from ADAM plasmids was produced by amplification of genomic DNA using primers that paired with the CRJE and C2 regions, only the data from the genome project was used to assess sequence conservation in these regions.)

Sequence uniformity in the CRJE was not quite absolute. Two sequence reads, which were identical to one another, contained an A residue at position 8 instead of the canonical G residue seen in other copies of the CRJE (See group 29 in Additional file 2). The polymorphism at position 8 alters the encoded peptide. Variation in the CRJE has been seen before, but remains quite rare [55].

**Figure 6:**
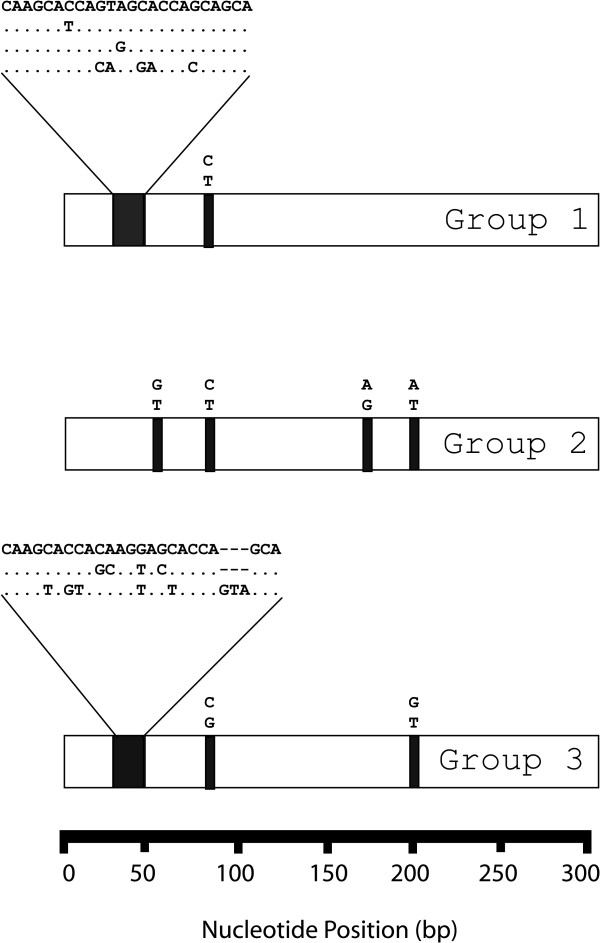
**Location and types of variation exhibited by closely-related sequence reads**. Data derived from the top three groups of sequence reads (**Table 1**) are shown. An open bar represents a group of aligned sequences. The black boxes within an open bar indicate the locations where variable bases were observed among the reads in the group. The sequences observed are shown above each black box. Dots represent identity.

Five hyper-variable regions are depicted in Figure 5. All of the hyper-variable regions exhibited a relatively high frequency of base substitution. Some hypervariable regions also exhibited frequent and extensive insertions and deletions (INDELS). To illustrate, hypervariable region 1 (HV1) began at site 28, where INDELS were very common. After the INDEL region, 15 of the next 20 nucleotide sites exhibited very frequent substitution. The types and locations of the substitutions in region HV1 are shown in Table 2, which shows the 31 different HV1 sequences that were observed in the 42 groups. Figure 6 shows that most of the nucleotide variation in groups 1, 2 and 3 occurred in HV1. In addition, in nearly half of the groups with 10 or more reads, HV1 variation was observed among the sequence reads in the group (Table 1).

**Table 2:** HV1 types observed in groups described in Table 1.

HV1 type	Nucleotide sequence of HV1 and encoded peptide
1	CAAGCACCAGTAGCACCAGCAGCA Q A P V A P A A

2	CAAGCATCAGGAGCACCAGCAGCA Q A S G A P A A

3	CAAGCACCAGTGGCACCAGCAGCA Q A P V A P A A

4	CAAGCACCACAAGGAGCACCAGCA Q A P Q G A P A

5	CAAGCACCAGCAGTACCACCAGCA Q A P A V P P A

6	CAAGTAGTACAAGTAGTACAAGTAGCA Q V V Q V V Q V A

7	CAAGCACCAGCAGGAGTAGTACAAGTAGCACAA Q A P A G V V Q V A Q

8	CAAGCACCAGCAGGAGTAGTACAAGGAGCACAA Q A P A G V V Q V A Q

9	CAAGCACAAGTAGTACAAGGAGCACAA Q A Q V V Q G A Q

10	CAAGCACAAGGAGTACAAGGAGCACAA Q A Q G V Q G A Q

11	CAAGCATTAGGAGTACAA Q A L G V Q

12	CAAGCACAAGGAGGAGCAGCAGCAGGAGCACAA Q A Q G G A A A G A Q

13	CAAGCAAAAGTAGTACAAGGAGCACAAGTAGCACAA Q A K V V Q G A Q V A Q

14	CAAGCACCACAAGCAGCACCAGCAGCG Q A P Q A A P A A

15	CAAAGACCACAAGGAGCACCAGCA Q R P Q G A P A

16	CAAGCAGTACAAGGAGCACAA Q A V Q G A Q

17	CAACAACAAGTGGCACAAGTAGCACAA Q Q Q V A Q V A Q

18	CAAGCAGCAGGAGGAGCAGCG Q A A G G A A

19	CAAGCAGCAGCACAAAAACAA Q A A A Q K Q

20	CGGGCGCGAGGAGTACAAGGAGCACAA R A R G V Q G A Q

21	CAAGCACAAGGAGCAGGAGGAGCAGCG Q A Q G A G G A A

22	CAAGCAGTACAAGGAGCAGCG Q A V Q G A A

23	CAAGCAGTACAAGGAGCAGTG Q A V Q G A V

24	CAAGCACAAGTAGTACAAAAACAA Q A Q V V Q K Q

25	CAAGCACAAGTAGTACAAGTAGCACAA Q A Q V V Q V A Q

26	CAAGCAGCAGTAGTAGCG Q A A V V A

27	CAAGCAGTAGGAGTAGTAGCG Q A V G V V A

28	CAAGCAGCAGGAGCAGTAGCG Q A A G A V A

29	CAAAATCAAGCAGCACCAGCAGCG Q N Q A A P A A

30	CAAGCAGCAGGAGTAGTAGCG Q A A G V V A

31	CAACAACAAGTACCACAAGGAGCACAA Q Q Q V P Q G A Q

### Functional significance of nucleotide variation

To assess the functional significance of the nucleotide differences that separate different MSG genes, the 73 core sequences were translated *in silico*. All but 6 of the core sequences contained an open reading frame encoding an MSG polypeptide. These 67 translationally competent sequences were aligned using RevTrans , which is a program that takes a set of DNA sequences, virtually translates them, aligns the peptide sequences, and uses this alignment as a scaffold for constructing the alignment of the corresponding DNA sequences [87]. Sites that did not have the same nucleotide in all 67 DNA sequences were scored. The changes seen at such sites were categorized as either nonsynonymous or synonymous, based on whether or not a nucleotide change caused a change in the encoded peptide sequence. The number of synonymous and non-synonymous substitutions and of insertions and deletions were calculated using a program called SNAP [88].

**Figure 7:**
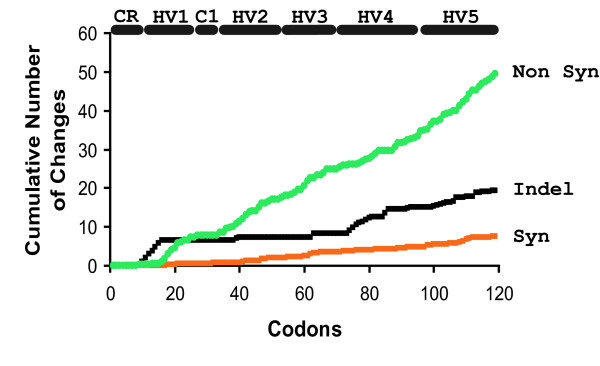
**Positive selection in MSG genes**. MSG genes were aligned and the frequency of non synonymous (abbreviated Non syn) and synonymous (Syn) substitutions as well as INDELS was scored for each codon. The horizontal lines labeled HV1 etc, demarcate five hypervariable regions. The horizontal lines labeled CR and C1 demarcate constant regions CRJE and C1.

Figure 7 plots the cumulative number of differences that occurred starting at the first codon and proceeding to the last. As would be expected from the data shown in Figure 5, INDELS and base substitutions occurred at multiple, but nonrandom locations. Variation was quite rare in the CRJE and C1 regions, and particularly frequent in HV regions. Occurrence of INDELS was strikingly frequent in HV1 and HV4.

### Role of selection in diversification of MSG gene family members

Statistical analysis of variation among the 67 translationally competent core MSG sequences showed that the rates of synonymous and nonsynonymous variation were significantly different (p = 0.0006, see Methods). In addition, the divergence seen in 48% of the 2211 pairwise comparisons of the 67 translationally competent core sequences appeared to have been influenced by positive selection for variation (p < 0.05, Z-test in MEGA 4.0). In Figure 7, the line describing non-synonymous differences increases nearly seven times faster than the line describing synonymous differences. This trend is the opposite of what is seen in most genes, where synonymous nucleotide substitutions tend to occur more frequently than non-synonymous substitutions. The prevalence of non-synonymous substitutions suggests that the MSG gene family has evolved under the influence of selection in favor of variation (positive selection). Positive selection has long been recognized as an important force in the evolution of both infectious organisms and defenses against them. An example is the Major Histocompatibility Complex (MHC), which facilitates the immune response against infectious microorganisms. There is a abundance of non-synonymous variation in the regions of MHC genes that encode the domains that bind microbial antigens [89].

### Recombination in MSG genes

**Figure 8:**
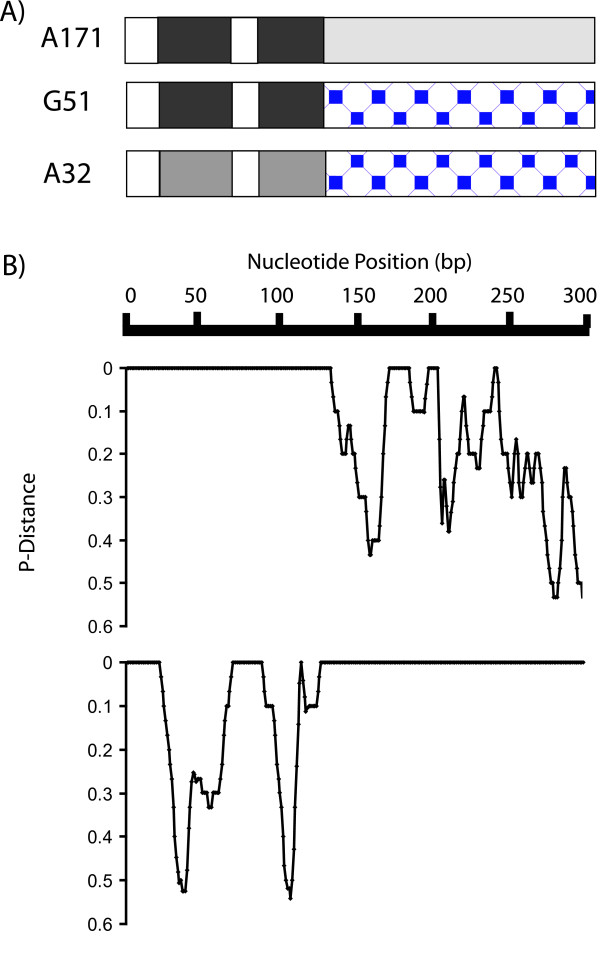
**Examples of gene structures suggestive of recombination between MSG genes**. A171, G51 and A32 are three MSG sequences. A) Box diagram showing regions of identity among the three sequences. Regions that are identical for at least 16 basepairs are the same shade and pattern. B) Plots of identity (indicated by p-Distance = 0) and non-identity (indicated by p-Distances greater than zero) in pair-wise alignments of sequences G51 and A171 (upper) and G51 and A32 (lower). P-distances were calculated using a window size of 10 nucleotides and a step size of 1 nucleotide.

Members of the MSG gene family share patches of sequence identity, which would be expected to facilitate homologous recombination between family members. Therefore, it might be expected that MSG genes would tend to recombine with each other. The possibility of recombination was examined in three ways. First the 73 sequences were analyzed with RDP2 software [90], which showed that each of the 73 MSG sequences exhibited evidence of recombination with at least one of the other 73 MSG sequences. Figure 8 shows an example of evidence suggestive of recombination. Sequences G51 and A171 were identical between positions 1 and 135 but not downstream of this region, where sequence G51 was identical to sequence A32. Sequences G51 and A32 were not identical in the region shared by sequences G51 and A171. Recombination between MSG genes would seem to be the most likely cause of the blocks of sequence identity observed when these three sequences were compared.

Recombination was also implicated by analysis of the HV1 region. The 42 consensus sequences contained 31 different sequences in the HV1 region. Therefore, some HV1 sequences were present in more than one gene. For example, HV1 sequence 4 was present in seven groups. Given that the HV1 region is so variable that it tends to differ in the different alleles of a gene, the presence of the same HV1 sequence in multiple genes would seem to not be due to sequence conservation, but rather be due to recombination events.

As a third test for recombination, subsections of the 73 MSG genes were analyzed. To start this analysis, each of the 73 sequences was divided into three segments, a, b and c, which corresponded to nucleotide positions 1 to 105, 106 to 210 and 211 to 315, respectively. Then, segments were aligned to the others of their type, i.e., a's were aligned to a's, etc. Three neighbor-joining trees, one for each segment, were made. Comparison of the three trees revealed many examples where a particular segment sequence occurred in more than one of the 73 MSG sequences. For example, one 'a' segment sequence occurred in nine different genes, which were different by virtue of the sequence in regions b and c. Recombination is the most parsimonious explanation for such gene structures.

PCR can generate recombinants [91-93]. To evaluate the possibility that this type of error contributed to the data, two plasmids, each carrying a distinct MSG insert (the two inserts were 80% identical), were mixed together and subjected to PCR amplification. The amplicons were cloned and twelve clones were picked and sequenced. One of these 12 cloned plasmids contained a recombinant insert. To determine if this result was indicative of the frequency of recombination, an additional 127 clones were screened for recombination by DNA hybridization using insert-specific oligonucleotide probes. All of the clones hybridized to one of the two probes utilized, but none hybridized to both. Therefore, only one recombinant was present among 139 plasmid clones tested, suggesting that the rate at which PCR produced recombinants was approximately 0.007. At this rate, it is improbable (p < 0.05) that more than one of the apparent recombinants observed among the 42 MSG sequences examined was created during PCR. In addition, any given artifactual recombination event would be relatively rare, and a sequence that is observed multiple times is unlikely to be an artifact. The three presumptive recombinants depicted in Figure 8 were seen in multiple sequence reads. Sequences A32, G51 and A171 were seen 40, 3 and 2 times, respectively.

## Discussion

The relationship between the 281 non-identical MSG sequences observed and the MSG gene family is subject to interpretation. The 281 sequences do not represent 281 different MSG genes because several lines of evidence indicate that there are only about 80 MSG genes in the genome. These lines of evidence include the results of previous quantitative hybridization experiments [50,94] and the results of quantitative PCR experiments described herein. The 80-gene family model is also supported by analysis of telomeric regions, where MSG genes are clustered. There are 17 *P. carinii *chromosomes each with two telomeres. All 34 telomeres were observed to hybridize to an MSG probe suggesting that there is at least one MSG gene per chromosome end. If so, there must be at least 34 MSG genes in the genome [54]. Sequence data obtained from 6 large telomeric DNA segments cloned into cosmid vectors showed that the average number of MSG genes per sequenced chromosome end was 2.2 [55]. If the other 28 telomeres, which were not present in the cosmid library, adhere to the structures observed in the 6 cosmid clones, then the *P. carinii *genome contains approximately 75 MSG genes. While the exact number of MSG gene at the ends of 28 telomeres is not known, each of these locations in the genome appears to contain approximately the same number of MSG genes because all telomeric fragments emitted similar amounts of radiation after having been hybridized to a radioactive MSG probe [54].

Removing reads that could have contained an error reduced the number of different sequences to from 281 to 158, a number approximately 2 times larger than the estimated size of the gene family. Allelic variation would explain this apparent excess of sequence diversity. Allelic variation in *P. carinii *has been described before, but seems to be fairly rare among unique genes [95-98]. However, allelic variation would be expected to be more common in genes that are members of a family that confers the capacity to vary surface antigens at a high rate. Allelic variation would also explain the presence of majority and minority sequences in groups of sequences, and fits with the disproportionate presence of these sequences in different populations of *P. carinii*.

Grouping sequence reads that were 95% or more identical produced a set of 73 sequences that would appear to represent most if not all of the genes in the MSG family. This proposition is supported by three arguments.

First, the set of 73 sequences contained 13 of 13 previously obtained *P. carinii *MSG gene sequences [51,55]. Obtaining this result would be improbable if the genome were to contain more than 91 different MSG gene sequences (p ≤ 0.047). However, if the gene copy-number is set at 77, then the observed result is ten times more probable (p = 0.5) (see Methods). Similarly, the proposition that the 73 core sequences describe all or nearly all of the MSG genes in the *P. carinii *genome was also supported by mark-recapture analysis [99], which produces an estimate of a gene family size from the number of sequences that are seen in two independently derived samples, which in this case were the ADAM and Lucigen libraries. The mark-recapture calculation suggested that the MSG gene family contains 85 genes. It should be noted, however, that the mark-recapture approach assumes that each MSG gene was equally likely to be observed. In fact, some MSG genes were observed numerous times while others were observed only once. The unevenness in the coverage of the gene family poses a major caveat when interpreting the accuracy of the results obtained with the mark-recapture analysis.

The second argument in favor of the proposition that the 73 sequences comprise a fairly complete picture of the family is that the methods used to produce the sequence reads would be expected to capture DNA segments from all MSG genes. The inserts in the plasmid libraries were generated by PCR that relied on priming from sequences (CRJE and C2) that are conserved among members of the gene family. By definition, MSG genes begin with a copy of the CRJE. Therefore, priming from the CRJE should amplify all MSG genes that contain a copy of the C2 region. Lack of the C2 region from one or more MSG gene is theoretically possible. However, analysis of sequences in the genome project database showed that the C2 primer binding site was present in all 448 sequence reads that contained the CRJE and were long enough to contain the C2 region. Therefore, if the genome contains MSG genes that would fail to be amplified by the primers employed, there are very few such genes. Even if the reliance on the presence of the C2 region had caused some genes to be excluded from the ADAM plasmid library, the genome project database would not be expected to be similarly deficient because this database was obtained by methods that did not rely on sequence conservation.

The third argument supporting the proposition that the 73 sequences comprise a fairly complete picture of the family is that the database of 581 sequence reads would seem to be large enough to virtually assure that all members of the family would be represented within it. Assuming that the gene family contains 80 genes, then the calculated probability of observing all family members by sampling 581 times is approximately 0.999. The exact size of the *P. carinii *MSG gene family is not known, but quantitative PCR experiments indicated that it contains no more than 90 genes. If there are 90 genes in the family, then the calculated probability of observing all family members by sampling 581 times is still very high, approximately 0.998. While the number of sequence reads obtained would seem to have been sufficient to produce a complete picture of the family, a caveat to this proposition arises from uncertainty about sample completeness. Sampling an 80-gene family 581 times should cover the family an average of 7 times. Therefore, the average coverage per gene should be approximately 7 fold. Yet, 31 of the different sequences in the set of 73 were seen only once, and the 10 largest groups of reads contained between 19 and 49 sequence reads each. These data depart significantly from expectations based on probability, which predict that no more than three of the genes would be represented less than twice or more than 14 times. Cloning bias is the probable cause of this deviation from expectation. It is commonly observed that some parts of genomes are relatively resistant to cloning in bacterial vectors. Another factor that probably contributed to the observed unevenness in coverage was that technical problems made it necessary to pursue the Pneumocystis genome project in a manner that would not be expected to produce equal coverage of the genome (See Methods). Whatever caused it, uneven coverage of the gene family would increase the chance of missing genes. New sequencing techniques that do not rely on cloning should help in addressing the possibility of missing data in the set of 73 sequences. Although control experiments showed that mis-incorporation of nucleotides and recombination during PCR are rare enough to discount these events as significant sources of the 31 sequences that were seen only once, the best way to confirm that a sequence is correct is to show that it can be reproducibly obtained. Therefore, more sequence data will be needed to confirm that the 31 singleton sequences are correct.

The set of 73 core MSG sequences contained three regions (CRJE, C1 and C2) where there was little to no variation in at least 16 contiguous nucleotide positions. Several other smaller regions (between 9 and 15 bp in length) also exhibited no variation among the 73 sequences. Conservation of these sequence elements cannot be ascribed to selection against changes in the encoded primary protein sequence because such selection would not prevent synonymous variation. Therefore, it would appear that the conserved nucleotides perform a function separate from and in addition to encoding an amino acid.

One possible function of conserved DNA sequence elements is to facilitate DNA recombination. Data described herein and previous studies indicate that donor MSG genes have recombined with each other [55,59,84]. Conserved sequence elements facilitate the DNA strand exchanges that occur during recombination. [100]. Conserved DNA sequence elements can also serve as targets for a site-specific nucleases, which generate a double stranded gap that is repaired via recombination with a related DNA sequences [101]. Such repairs are often called gene conversions because the DNA that fills the gap is copied from donor DNA while it is being inserted. Therefore the donor DNA remains unchanged. Sometimes gap repair also causes crossing-over [102]. *Saccharomyces cerevisiae *has several such nucleases including HO, Spo11 and I-Sce1 [103-106]. HO and Spo11 function in controlling mating type and meiotic recombination, respectively. Therefore, these types of enzymes are needed to perform fundamental functions, and it would not be surprising if *P. carinii *possesses site-specific nucleases. Another possible role for conserved sequence elements would be as sites for site-specific recombinases, which are enzymes that catalyze crossovers by facilitating breakage and reunion between two copies of the site recognized by the recombinase [107].

Comparing the MSG genes at the expression site to donor MSG genes might allow assessment of the roles of crossing-over and gene conversion in creating the sequence diversity at the expression site. For example, if the gene at the expression site were installed there by a reciprocal cross-over, then the gene that is at the expression site will have vacated its former locus and been replaced by the gene that was formerly at the expression site. On the other hand, if gene conversion causes a change at the expression site, then the same sequence will exist at both the expression site and the donor site.

While high conservation of nucleotide tracts in MSG genes might be needed to foster recombination, such conservation causes high conservation in certain parts of MSG proteins, which would seem problematic if the function of MSGs is to impart surface variation. However, the functional ramifications of diverse MSG proteins containing invariant regions will be influenced by other factors, such post-translational processing and three-dimensional structure. The region encoded by the UCS and CRJE appears to be present on the MSG proteins when they are first produced, but not present on MSG proteins on the cell surface [94]. The protein segments encoded by the C1 and C2 regions are rich in hydrophobic amino acids and are probably buried within the MSG molecule, it which case, they would be unlikely to be detected by the host immune system.

The nucleotides located between conserved regions varied to different degrees. The HV1 region was the most clearly delineated, being located between the CRJE and C1, which are both very highly conserved. Other hypervariable regions had less distinct borders. Sequence variation included base substitutions and INDELS. Substitutions often were nonsynonymous, a finding that suggests that selection has favored changes that change the MSG protein encoded [108]. Selection for variation is consistent with the idea that MSG genes confer the ability to deploy protective antigenic variation. High frequency protein-changing nucleotide variation has been observed to extend beyond the first 325 bps of MSG genes, and probably does so in most if not all MSG genes. Previous analysis of 11 complete MSG genes showed that they varied throughout their length and that positive selection caused much of this variation [55].

INDELS did not often alter the reading frame because the number of nucleotides involved in nearly all INDELS was divisible by 3. It is interesting to note that despite the great variation seen among the 581 sequence reads, nearly all of these reads contained an open reading frame encoding an MSG protein. These findings suggest that the entire gene family is under selective pressure to continue encoding intact MSG proteins that differ one from another.

Some of the regions in which INDELS were prevalent contained simple repeats, such as short run of A:T base pairs. Such mononucleotide repeats are more prone to change in size due to slippage of DNA polymerase during genome replication, repair and recombination [109]. However, most of the INDELS involved more complex sequences that did not appear to have been formed via interactions between short identical repeats.

## Conclusion

A set of sequences that represents most if not all of the members of the *P. carinii *MSG gene family was obtained. The protein-changing nature of the variation among these sequences suggests that the family has been shaped by selection for protein variation, which is consistent with the hypothesis that the MSG gene family functions to enhance phenotypic variation among the members of a population of *P. carinii*.

Understanding the *P. carinii *MSG gene family at the sequence level provides an avenue through which to assess the function of this family, which is currently unknown, but presumably contributes to the ability of the fungus to parasitize its host, *Rattus norwegicus*. With the improved understanding of the family provided by the studies described herein, it should be possible to determine if changes at the expression site involve gene conversions, crossovers, or both. It should also be possible to learn more about the rate and mechanism of evolution of MSG genes, which would be expected to be occurring rapidly via selection for mutation and recombination.

## Methods

### Construction of plasmid libraries

Two types of plasmid libraries containing DNA from *P. carinii *MSG genes were generated. One type of library (called ADAM) was made by cloning MSG gene DNA that had been specifically amplified from genomic *P. carinii *DNA. All of the ADAM plasmid clones contained inserts that were made by amplifying genomic DNA using a primer that binds to the CRJE, which marks the 5-prime end of every MSG gene. The vast majority of plasmid inserts contained segment that had been amplified using primer C2 as the downstream primer. Primer C2 binds to the C2 sequence, which is conserved among family members and located approximately 300 basepairs downstream of CRJE [66,69]. Additional libraries were made by amplifying genomic DNA using the CRJE primer paired with either primer C5 or C7, which bind to conserved sequences located downstream of the C2 sequence [55]. Primers are listed in Additional file 3. The PCR products were inserted into the plasmid vector TOPO 2.1 (Invitrogen, Carlsbad, CA). The plasmids were introduced into *E. coli *strain TOP10 by standard methods [110].

The second type of plasmid library was made by Lucigen Corporation [70,111]. The *P. carinii *genomic DNA used to prepare Lucigen libraries was obtained from *P. carinii *chromosomes that had been separated from each other, and from most contaminants, via pulsed field gel electrophoresis [73,79,112]. After electrophoresis, either individual or small groups of *P. carinii *chromosomes were extracted from the gel and used to create a number of different plasmid libraries [70,111]. The use of pulsed-field gels to obtain *P. carinii *chromosomes was necessary because the *P. carinii *used had been prepared from the lungs of immunosuppressed infected rats, and contained rat cells and DNA as well as assorted other microbes. The use of rat lung was necessitated by the lack of sufficient proliferation of *P. carinii *in culture. Shotgun cloning from impure *P. carinii *DNA turned out to be very inefficient because *P. carinii *DNA, which is rich in A:T basepairs, competed poorly with DNA from the rat and from other microbes coexisting with *P. carinii *in the infected rats. The problem of cloning bias against *P. carinii *DNA was ameliorated by purifying *P. carinii *chromosomes.

Processing of the DNA from *P. carinii *chromosomes was described by Sesterhenn et. al. [70,111]. Briefly, the DNA was processed, either by restriction enzyme digestion or sonication, to reduce it to an average size of approximately 2 kb [70,111]. DNA fragments were ligated to oligonucleotides that served as sites for PCR primers. DNA segments produced by PCR amplification were ligated to the Lucigen pSMART-HCKan cloning vector. Several Lucigen libraries were made, each from the fraction of the *P. carinii *genome contained in the chromosome or set of chromosomes excised from a region of a pulsed field gel. Different libraries contained different numbers of clones, and the number of clones was not necessarily proportional to the number of basepairs of *P. carinii *genomic DNA that was in a given chromosome or set of chromosomes. This situation predicted that coverage of the MSG gene family would be uneven, and that some MSG genes would be more frequently observed in the sequence reads in the genome project database.

### Obtaining and identifying MSG gene sequences

The inserts in 247 plasmids from the ADAM libraries were sequenced by commercial sequencing facilities. Sequences obtained from the ADAM plasmid libraries were used to identify MSG reads in the Pneumocystis genome project sequence database, nearly all of which had been derived from Lucigen libraries. First, the ADAM sequence reads were assembled using Gap4 software [113] set at 1% maximum mismatch. This process identified 73 different sequences. These sequences were used to perform an *in silico *search of the Pneumocystis genome project sequence database. The 1156 sequence reads that were 90% or more identical to one or more of the 73 ADAM sequences were placed in a database for further analysis. The 1156 sequence reads contained 99.7% of the CRJE-containing sequences in the Pneumocystis genome project sequence database. A perl script  that implemented Dynamic Programming was utilized to identify which of the 1156 sequence reads contained both the CRJE sequence and the C2 region [114]. The perl script also truncated each read at the 5' terminus of the CRJE and oriented the reads in a 5' to 3' direction. The sequence reads that contained no more than 3 substitutions in the CRJE were placed into a database. (Excluding sequences with a CRJE sequence that was more divergent was done in order to exclude any sequences that may have come from *P. wakefieldiae*, which can co-infect rats that are infected with *P. carinii. P. wakefieldiae *MSG genes start with a CRJE that differs from that in *P. carinii *CRJE at 5 positions [75].) Some of the data in the genome project database had been entered more than once. Duplicate entries were removed. Also removed were sister reads and reads containing ambiguous base calls. These procedures left 334 high-quality sequence reads that contained the CRJE and the C2 sequence, and were at least 90% identical to one or more ADAM sequences. Combining the 334 genome project sequence reads with the 247 ADAM sequences provided 581 sequences for comparative analysis, which is more than 7 times the number of genes in the *P. carinii *MSG gene family, which contains approximately 80 genes [50,69,79,80].

### Sequence alignment and comparison

The 581 sequence reads were assembled with Gap4 software [113]. The following software settings were utilized: minimum initial match was 20, 25 maximum pads per read, and maximum percent mismatch of 5. To confirm the results obtained with Gap4, the sequence reads were also assembled with Cap3 [115]. Cap3 default settings were used except the overlap percent identity cutoff was set at 95%. DNA sequences were aligned using MAFFT software using default settings [116]. The alignments were optimized by introducing a limited number of gaps. The relatedness of sequences was evaluated and depicted using Mega 4.0 software [117].

The 581 sequence reads contained many reads that were identical to one or more other reads. Removing duplicate reads reduced the size of the database to 281. A downloadable file containing all 281 MSG sequences can be found at .

### Assessing the influence of selection

The 281 sequence reads contained 123 reads that differed from other reads at only one position. Given that such minimal divergence could have been due to error, these sequences were grouped with their close relatives, which left 158 reads that differed at more than one site. Analysis of the 158 reads showed that all but 31 of them could be grouped into one of 42 groups, the members of which differed by no more than five percent, which could have been due to allelic variation. To simplify analysis of the influence of selection on the gene family, consensus sequence was derived from each of the 42 groups. Combining the 42 consensus sequences with the 31 singleton sequences produced a core set of 73 sequences, 67 of which contained a single open reading frame (ORF) encoding an MSG polypeptide. The 73 sequences can be downloaded at . The 67 DNA sequences with a single open reading frame were aligned with the aid of RevTrans software [87], which is a program that takes a set of DNA sequences, virtually translates them, aligns the peptide sequences, and uses this alignment as a scaffold for constructing the alignment of the corresponding DNA sequences. The numbers of synonymous substitutions, non-synonymous substitutions, insertions and deletions were calculated using SNAP [88].

The software package MEGA 4.0 was used to assess the role of selection in the substitutions seen among MSG genes [117,118]. All possible pairs of sequences were analyzed. The average number of synonymous substitutions (dS) and the average number of nonsynonymous substitutions (dN) were obtained (gaps were ignored). The variance of the difference between dS and dN was estimated by the bootstrap method using 500 replicates. The Z-test statistic was obtained from the following: Z = (dN - dS)/SQRT(Var(dS) + Var(dN)).

### Tests for recombination

The 67 sequences with an MSG-encoding ORF were aligned with Bioedit software [119]. Recombination tests were performed with RDP2 software [90], which uses probabilistic tests to identify probable recombinants. In addition, each of the 73 core sequences was divided into 3 segments, a, b and c. The "a" segments were aligned to each other, as were the b and c segments. Neighbor-joining trees were constructed for each segment using MEGA 4.0 software. To assess recombination, the trees were examined for cases where different genes were identical in one or two segments. Extensive local identity within regions that vary globally is indicative of recombination.

### Control experiments to assess recombination during PCR

To determine if recombination occurred during PCR, two plasmids carrying different MSG genes (A and O) were mixed together and subjected to PCR. PCR was performed under the following conditions: 94°C hot start for 3 minutes, 20 (for 1 × 10^6 ^copies) or 30 cycles (for 2 × 10^4 ^copies) of incubation at 94°C for 60 sec, 55°C for 120 sec and 72°C for 60 sec followed by 1 cycle of 72°C for 10 minutes. Reaction volumes were 25 ul containing 100 uM each of dATP, dCTP, dGTP, dTTP, 1 U of *Taq *polymerase (Promega, Madison WI), 2.5 mM MgCl_2_, and 50 ng each of IG1 and C6 primers (see Additional file 3). The amplicons produced were approximately 1300 bp in size, as expected. The amplicons were inserted into the TOPO 4.0 plasmid vector (Invitrogen, Carlsbad, CA) and the plasmids were introduced into *E. coli *strain TOP10 by the One Shot Chemical Transformation Protocol as described in the TOPO TA cloning kit manual (Invitrogen). To detect colonies with recombinant plasmid inserts, a DNA hybridization screen was employed. Colonies to be screened were picked and arrayed on Hybond-N membranes (Amersham Biosciences, Buckinghamshire UK) that were sitting on a surface of nutrient agar [110]. The agar plates and inoculated membranes were incubated overnight at 37°C to allow colonies to expand. To prepare a "colony blot", plasmid DNA was released from bacterial colonies and fixed to the membrane by standard methods employing alkaline lysis, neutralization and desiccation in high salt [110]. Radioactive oligonucleotides to be used as probes were labeled with T4 polynucleotide kinase (Invitrogen) [110]. The four oligonucleotides employed are listed in Additional file 3. Oligonucleotides A1 and A2 were specific for the MSG A gene sequence, while oligonucleotides O1 and O2 were specific for the MSG O gene sequence.

Hybridization was performed in Rapid-Hyb (GE Healthcare) at 42°C for 18 hours. After hybridization, membranes were washed at room temperature for 30 minutes in a buffer containing 6× SSC (0.9 M NaCl, 0.09 M NaCitrate) and 0.1% sodium dodecyl sulfate (SDS), and then washed for 1 hour in a buffer containing 3 M tetramethyammonium chloride, 50 mM Tris/HCl pH 8·0, 2 mM EDTA, 0·1% SDS [120] utilizing the temperatures listed in Additional file 3. Bound radioactive probe was detected by autoradiography. As a positive control, each colony blot contained a colony carrying a plasmid designed to hybridize to all probes.

### Quantification of the number of MSG loci in the genome by real-time PCR

To measure the number of MSG loci in the *P. carinii *genome, genomic DNA was analyzed by real-time PCR performed in a Cepheid Smart Cycler using Smart Cycler software version 1.2d (Sunnyvale, CA, USA). PCR was performed under the following conditions: 95°C hot start for 120 seconds, 40 cycles of incubation at 95°C for 15 sec, 51°C for 15 sec, 72°C for 15 sec, and 80°C for 10 seconds with optics set to detect SYBR Green fluorescence. Reaction volumes were 25 ul containing 100 uM each of dATP, dCTP, dGTP, dTTP, 3 U of *Tfl *polymerase (Epicenter), 5 mM MgCl_2_, 1 μl of a 1:20,000 dilution of SYBR Green I (BioWhitaker Molecular Applications), 20 ng of each primer and approximately 1 microgram of *P. carinii *DNA. Primers CRJE-RT and C1 (Additional file 3) were used in reactions designed to amplify all of the MSG genes in the genome. Primers -145 and anti-AUG [69] were used in reactions designed to amplify the UCS, under the same conditions given above except the annealing temperature used was 45°C. Amplification was monitored by the increase in SYBR Green fluorescence.

The amount of the target DNA in a given PCR tube was inferred from the number of cycles required to reach a threshold level of SYBR Green fluorescence. A standard curve relating threshold cycle to DNA amount was produced by observing the number of cycles required to reach a threshold level of SYBR Green fluorescence in reactions containing known amounts of a plasmid carrying an insert that amplified with both pairs of primers.

### Assessment of the completeness of the set of 73 core sequences

If the set of 73 core sequences contains all MSG genes, then every MSG gene sequence drawn from any other source will be found in the 73. If the set of 73 core sequences does not contain all MSG genes, then there will be a probability that a given subset of MSG genes sequences drawn from another source will be found in the 73. The probability of observing the subset will decline as the sizes of the gene family and subset increase. Therefore, one can assess the completeness of the set of 73 core sequences by determining if it contains some or all of a previously described set of MSG genes. When the set of 73 was searched for the sequences from 13 previously described MSG genes [51,55], all 13 were found. This result was consistent with the proposition that the set of 73 contains all MSG genes. To evaluate the chance that such a result would occur even if the genome were to contain more than 73 MSG genes, the relationship between the chance of observing all 13 of the previously known MSG genes, and the actual size of the gene family was assessed as follows. If the actual size of the MSG gene family is X, then the probability that the set of 73 genes will contain a given family member is obtained by dividing 73 by X. For example, if there are 80 genes in the family, then the probability of observing any particular gene in the set of 73 is 73/80 = 0.9125. The probability of observing all 13 of the 13 known MSG genes is (0.9125)^13 ^= 0.3. Therefore, the hypothesis that the gene family contains 80 members is consistent with the fact that all 13 genes were present in the core set of 73 MSG sequences. By contrast, when the size of the gene family is set at 92, the probability of observing all of the 13 known MSG genes is 0.047. When the size of the gene family is set at 158, the probability of observing the 13 known MSG genes is only 4 × 10^-5^.

### Mark-recapture method

The total number of MSG genes was also estimated using a modified Petersen-Lincoln estimator [99]. The method uses data from two independent samples drawn from the same population. In this case, the *P. carinii *genome was the population, and the two samples were the sequences from the ADAM and Lucigen libraries. In brief, the estimator used the number of different sequences in each sample, and the number of sequences seen in both samples, to calculate the probable the size of the gene family.

## Abbreviations

CRJE: Conserved Recombination Junction Element; MSG: Major Surface Glycoprotein; MSR: MSG-related; UCS: Upstream Conserved Sequence; HV1: Hypervariable Region 1; VSG: Variable Surface Glycoprotein; INDELS: Insertions and/or deletions; Non syn: non synonymous; Syn: synonymous.

## Authors' contributions

SPK performed PCR, constructed libraries, acquired sequence data, performed in silico analysis of sequence data, performed statistical tests, interpreted results and helped write the manuscript. JRS performed statistical tests, interpreted results and helped write the manuscript. All authors read and approved the final version of the manuscript.

## Supplementary Material

Additional file 1 **Distribution of MSG sequence variation assessed by pair-wise comparisons of non-identical reads**. The 281 non-identical sequences were compared pairwise. For each pair of sequences, the number of differences was computed with MEGA4 software [117]. INDELS were ignored. Click here for file

Additional file 2 **Supplemental Table 1**. Frequencies of putative MSG alleles in five populations of *P. carinii*. Click here for file

Additional file 3 **Supplemental Table 2**. Oligonucleotides used in this study. Click here for file
