# Supplementary material for: Complexity of the MSG gene family of Pneumocystis carinii
Source: BMC Genomics. 2009 Aug 7;10:367. doi: 10.1186/1471-2164-10-367 (PMC2743713; doi:10.1186/1471-2164-10-367)
Supplement: Additional file 2 — Supplemental Table 1. Frequencies of putative MSG alleles in five populations of P. carinii. [file 1471-2164-10-367-S2.doc]

**Supplemental Table 1. Frequencies of putative MSG alleles in five populations of *P. carinii***

| Group^a^ (no. reads) | | Nucleotide  position of polymorphism^b^ | Haplotypes^c^ | | | Frequency of haplotypes  in 5 *P. carinii* populations ^d^ | | | | | | |
| --- | --- | --- | --- | --- | --- | --- | --- | --- | --- | --- | --- | --- |
|  | |  |  | | | A | | B | C | | D | E |
| 1(49) | | HV1,81 | 1-C | | | 15 | | 3 | 1 | | 1 | 5 |
|  | |  | 1-T | | | 0 | | 2 | 0 | | 0 | 0 |
|  | |  | 2-C | | | 4 | | 15 | 0 | | 0 | 1 |
|  | |  | 2-T | | | 0 | | 1 | 0 | | 0 | 0 |
|  | |  | 3-C | | | 1 | | 0 | 0 | | 0 | 0 |
|  | |  | 4-C | | | 1 | | 0 | 0 | | 0 | 0 |
|  | |  |  | | |  | |  |  | |  |  |
| 2 (40) | | 57,83,173,204 | 5-GTAA | | | 6 | | 24 | 0 | | 0 | 0 |
|  | |  | 5-TTAA | | | 0 | | 2 | 0 | | 0 | 0 |
|  | |  | 5-GCAA | | | 0 | | 2 | 0 | | 0 | 0 |
|  | |  | 5-GTGA | | | 4 | | 0 | 0 | | 0 | 0 |
|  | |  | 5-GTAT | | | 2 | | 0 | 0 | | 0 | 0 |
|  | |  |  | | |  | |  |  | |  |  |
| 3 (34) | | HV1,85,203 | 4-CT | | | 9 | | 16 | 1 | | 0 | 1 |
|  | |  | 4-CG | | | 1 | | 0 | 1 | | 0 | 0 |
|  | |  | 6-CT | | | 0 | | 0 | 0 | | 0 | 1 |
|  | |  | 6-CG | | | 2 | | 0 | 0 | | 0 | 0 |
|  | |  | 5-GG | | | 1 | | 0 | 1 | | 0 | 0 |
|  | |  |  | | |  | |  |  | |  |  |
| 4 (32) | | HV1 | 7 | | | 10 | | 16 | 1 | | 0 | 0 |
|  | |  | 8 | | | 2 | | 0 | 0 | | 0 | 0 |
|  | |  | 9 | | | 0 | | 0 | 1 | | 1 | 0 |
|  | |  | 10 | | | 1 | | 0 | 0 | | 0 | 0 |
|  | |  |  | | |  | |  |  | |  |  |
| 5 (29) | | 153 | 11-T | | | 5 | | 22 | 0 | | 0 | 0 |
|  | |  | 11-C | | | 0 | | 2 | 0 | | 0 | 0 |
|  | |  |  | | |  | |  |  | |  |  |
| 6 (28) | | HV1, 300 | 12-T | | | 4 | | 20 | 1 | | 0 | 0 |
|  | |  | 12-C | | | 0 | | 2 | 0 | | 0 | 0 |
|  | |  | 13-T | | | 1 | | 0 | 0 | | 0 | 0 |
|  | |  |  | | |  | |  |  | |  |  |
| 7 (26) | | 193 | 14-A | | | 2 | | 22 | 0 | | 0 | 0 |
|  | |  | 14-G | | | 0 | | 2 | 0 | | 0 | 0 |
|  | |  |  | | |  | |  |  | |  |  |
| 8 (24) | | HV1 | 9 | | | 13 | | 7 | 0 | | 0 | 1 |
|  | |  | 7 | | | 2 | | 0 | 0 | | 1 | 0 |
|  | |  |  | | |  | |  |  | |  |  |
|  | |  |  | | |  | |  |  | |  |  |
|  | |  |  | | |  | |  |  | |  |  |
|  | |  |  | | |  | |  |  | |  |  |
| **Supplemental Table 1. continued** | | | | | | | | | | | | |
| Group^a^ (no. reads) | | Nucleotide  position of polymorphism^b^ | | | Haplotypes^c^ | | Frequency of haplotypes  in 5 *P. carinii* populations ^d^ | | | | | |
|  | |  | | |  | | A | B | | C | D | E |
| 9 (25) | | HV1,302,313 | | | 4-AA | | 3 | 14 | | 0 | 0 | 0 |
|  | |  | | | 4-GA | | 0 | 4 | | 0 | 0 | 0 |
|  | |  | | | 4-AG | | 0 | 2 | | 0 | 0 | 0 |
|  | |  | | | 1-AA | | 1 | 0 | | 0 | 0 | 0 |
|  | |  | | | 15-AA | | 1 | 0 | | 0 | 0 | 0 |
|  | |  | | |  | |  |  | |  |  |  |
| 10 (19) | | 97 to 107 | | | 16 | | 8 | 10 | | 0 | 0 | 0 |
|  | |  | | | 16-indel | | 1 | 0 | | 0 | 0 | 0 |
|  | |  | | |  | |  |  | |  |  |  |
| 11 (19) | | NA | | | 17 | | 1 | 18 | | 0 | 0 | 0 |
|  | |  | | |  | |  |  | |  |  |  |
| 12 (18) | | 65,216,228,282 | | | 18-T-TA | | 2 | 11 | | 0 | 0 | 0 |
|  | |  | | | 18-A-TA | | 0 | 1 | | 0 | 0 | 0 |
|  | |  | | | 18-AATG | | 0 | 1 | | 0 | 0 | 0 |
|  | |  | | | 18-TATA | | 0 | 1 | | 0 | 0 | 0 |
|  | |  | | | 18-T-AA | | 0 | 2 | | 0 | 0 | 0 |
|  | |  | | |  | |  |  | |  |  |  |
| 13 (16) | | HV1 | | | 9 | | 1 | 0 | | 0 | 0 | 0 |
|  | |  | | | 16 | | 15 | 0 | | 0 | 0 | 0 |
|  | |  | | |  | |  |  | |  |  |  |
| 14 (13) | | NA | | | 19 | | 8 | 3 | | 2 | 0 | 0 |
|  | |  | | |  | |  |  | |  |  |  |
| 15 (13) | | HV1 | | | 10 | | 5 | 6 | | 1 | 0 | 0 |
|  | |  | | | 20 | | 1 | 0 | | 0 | 0 | 0 |
|  | |  | | |  | |  |  | |  |  |  |
| 16 (13) | | NA | | | 21 | | 5 | 7 | | 1 | 0 | 0 |
|  | |  | | |  | |  |  | |  |  |  |
| 17 (13) | | NA | | | 4 | | 8 | 5 | | 0 | 0 | 0 |
|  | |  | | |  | |  |  | |  |  |  |
| 18 (13) | | HV1 | | | 22 | | 0 | 10 | | 0 | 0 | 0 |
|  | |  | | | 23 | | 2 | 1 | | 0 | 0 | 0 |
|  | |  | | |  | |  |  | |  |  |  |
| 19 (11) | | NA | | | 16 | | 6 | 5 | | 0 | 0 | 0 |
|  | |  | | |  | |  |  | |  |  |  |
| 20 (11) | | NA | | | 16 | | 4 | 6 | | 1 | 0 | 0 |
|  | |  | | |  | |  |  | |  |  |  |
| 21 (10) | | 53,54,58,145 | | | 16-GTAA | | 2 | 0 | | 0 | 0 | 0 |
|  | |  | | | 16-AGGG | | 6 | 1 | | 1 | 0 | 0 |
|  | |  | | |  | |  |  | |  |  |  |
| 22 (9) | | NA | | | 5 | | 1 | 8 | | 0 | 0 | 0 |
| **Supplemental Table 1. continued** | | | | | | | | | | | | |
| Group^a^ (no. reads) | Nucleotide  position of polymorphism^b^ | | | Haplotypes^c^ | | | Frequency of haplotypes  in 5 *P. carinii* populations ^d^ | | | | | |
|  |  | | |  | | | A | B | | C | D | E |
| 23 (9) | 106 | | | 24-G | | | 0 | 7 | | 0 | 0 | 0 |
|  |  | | | 24-A | | | 0 | 2 | | 0 | 0 | 0 |
|  |  | | |  | | |  |  | |  |  |  |
| 24 (8) | 80 | | | 25-T | | | 4 | 2 | | 0 | 0 | 0 |
|  |  | | | 25-C | | | 0 | 2 | | 0 | 0 | 0 |
|  |  | | |  | | |  |  | |  |  |  |
| 25 (7) | NA | | | 25 | | | 5 | 2 | | 0 | 0 | 0 |
|  |  | | |  | | |  |  | |  |  |  |
| 26 (7) | HV1 | | | 26 | | | 1 | 3 | | 0 | 0 | 0 |
|  |  | | | 27 | | | 0 | 3 | | 0 | 0 | 0 |
|  |  | | |  | | |  |  | |  |  |  |
| 27 (6) | NA | | | 28 | | | 1 | 5 | | 0 | 0 | 0 |
|  |  | | |  | | |  |  | |  |  |  |
| 28 (5) | NA | | | 16 | | | 2 | 3 | | 0 | 0 | 0 |
|  |  | | |  | | |  |  | |  |  |  |
| 29 (4) | 8 | | | 29-G | | | 0 | 2 | | 0 | 0 | 0 |
|  |  | | | 29-A | | | 0 | 2 | | 0 | 0 | 0 |
|  |  | | |  | | |  |  | |  |  |  |
| 30 (5) | HV1,296 | | | 19-T | | | 0 | 3 | | 0 | 0 | 0 |
|  |  | | | 24-C | | | 0 | 2 | | 0 | 0 | 0 |
|  |  | | |  | | |  |  | |  |  |  |
| 31 (4) | NA | | | 4 | | | 4 | 0 | | 0 | 0 | 0 |
|  |  | | |  | | |  |  | |  |  |  |
| 32 (4) | NA | | | 4 | | | 0 | 4 | | 0 | 0 | 0 |
|  |  | | |  | | |  |  | |  |  |  |
| 33 (3) | NA | | | 30 | | | 1 | 2 | | 0 | 0 | 0 |
|  |  | | |  | | |  |  | |  |  |  |
| 34 (3) | HV1 | | | 31 | | | 2 | 0 | | 0 | 0 | 0 |
|  |  | | | 16 | | | 1 | 0 | | 0 | 0 | 0 |
| 35 (3) | NA | | | 4 | | | 2 | 0 | | 1 | 0 | 0 |
|  |  | | |  | | |  |  | |  |  |  |
| 36 (3) | NA | | | 21 | | | 3 | 0 | | 0 | 0 | 0 |
|  |  | | |  | | |  |  | |  |  |  |
| 37 (3) | NA | | | 7 | | | 0 | 3 | | 0 | 0 | 0 |
|  |  | | |  | | |  |  | |  |  |  |
| 38 (3) | NA | | | 25 | | | 0 | 3 | | 0 | 0 | 0 |

^a^ 581 reads were assembled (maximum mismatch of 5%) into groups. Groups containing less than 3 reads are not listed.

^b^ “HV1” means that a polymorphism was seen in hypervariable region 1 (see Table 2 for sequences). Numbers in this column refer to the location of the polymorphisms that were not in HV1. Each number refers to a nucleotide site where position 1 is the A in the ATG at the beginning of the CRJE. Because the HV1 sequences varied in length, the position-numbers of polymorphisms downstream of HV1 cannot be compared between groups. NA: Not applicable because only one haplotype was seen.

^c^ An haplotype designated as 1-C had a type 1 hypervariable region and a C at a polymorphic site located outside of HV1.

^d^ Populations A and B were the source of ADAM plasmids and Lucigen genome project reads, respectively. Populations C, D and E were smaller populations that had been analyzed in the past using the same methods as those used to produce the ADAM plasmid library.
