# Supplementary material for: Complexity of the MSG gene family of Pneumocystis carinii
Source: BMC Genomics. 2009 Aug 7;10:367. doi: 10.1186/1471-2164-10-367 (PMC2743713; doi:10.1186/1471-2164-10-367)
Supplement: Additional file 3 — Supplemental Table 2. Oligonucleotides used in this study. [file 1471-2164-10-367-S3.doc]

Supplemental Table 2. Oligonucleotides used in this study.

| Name | Sequence (5’ to 3’) | Location  in MSG^a^ | Length | T (°C) | |
| --- | --- | --- | --- | --- | --- |
|  |  |  |  | Hyb^b^ | PCR^c^ |
| -145 | TAGACGATATGAAGGGAGAAT | -145 | 21 | NA | 45 |
| CRJE-RT | ATGGCACGGCCGGTTAA | +1 | 17 | NA^d^ | 51 |
| CRJE | ATGGCACGGCCGGTTAAGAG | +1 | 20 | 55 | 50 |
| α-CRJE | CTCTTAACCGGCCGTGCCAT | +20 | 20 | NA | 45 |
| IG1 | ATCCAACTTAGAAACTACCC | -45 | 20 | NA | 55 |
| C1 | AATRAAAGCCAAAAGGKGT | +80 | 19 | NA | 51 |
| C2 | ATACATTTTTCTTCATGTTTT | +318 | 20 | NA | 50 |
| C5 | CATGAAAGACTTGAGAAATGT | +628 | 21 | NA | 45 |
| C6 | CTTGAGTCGGAATGTTTCTATTTA | +1201 | 23 | NA | 55 |
| C7 | GTCTTGTCCCTTTTTATAGCA | +1288 | 22 | NA | 50 |
| A1 | AGCAGCAGCACAAAAACAAGATGA | +24 | 24 | 59 | NA |
| A2 | CATTCTGAAGAAGGAGTATAAAGATG | +78 | 26 | 59 | NA |
| O1 | CAACCCAATACTTCTTAACA | +797 | 20 | 55 | NA |
| O2 | ATAATGAAAAATCAGATCCT | +1115 | 19 | 55 | NA |

^a^Relative to the methionine start codon of donor MSG genes; + is downstream of start codon; - is upstream of start codon.

^b^Temperature used when the oligonucleotide was used as a hybridization probe.

^c^Temperature used when the oligonucleotide was used as a PCR primer.

^d^ NA-not applicable
